# Effects of a social accountability approach, CARE’s Community Score Card, on reproductive health-related outcomes in Malawi: A cluster-randomized controlled evaluation

**DOI:** 10.1371/journal.pone.0171316

**Published:** 2017-02-10

**Authors:** Sara Gullo, Christine Galavotti, Anne Sebert Kuhlmann, Thumbiko Msiska, Phil Hastings, C. Nathan Marti

**Affiliations:** 1 CARE USA, Atlanta, GA, United States of America; 2 College for Public Health & Social Justice, Saint Louis University, St. Louis, MO, United States of America; 3 CARE Malawi, Lilongwe, Malawi; 4 Far Harbor, LLC, Austin, TX, United States of America; National Institute of Health, ITALY

## Abstract

**Background:**

Social accountability approaches, which emphasize mutual responsibility and accountability by community members, health care workers, and local health officials for improving health outcomes in the community, are increasingly being employed in low-resource settings. We evaluated the effects of a social accountability approach, CARE’s Community Score Card (CSC), on reproductive health outcomes in Ntcheu district, Malawi using a cluster-randomized control design.

**Methods:**

We matched 10 pairs of communities, randomly assigning one from each pair to intervention and control arms. We conducted two independent cross-sectional surveys of women who had given birth in the last 12 months, at baseline and at two years post-baseline. Using difference-in-difference (DiD) and local average treatment effect (LATE) estimates, we evaluated the effects on outcomes including modern contraceptive use, antenatal and postnatal care service utilization, and service satisfaction. We also evaluated changes in indicators developed by community members and service providers in the intervention areas.

**Results:**

DiD analyses showed significantly greater improvements in the proportion of women receiving a home visit during pregnancy (*B* = 0.20, *P* < .01), receiving a postnatal visit (*B* = 0.06, *P* = .01), and overall service satisfaction (*B* = 0.16, *P* < .001) in intervention compared to control areas. LATE analyses estimated significant effects of the CSC intervention on home visits by health workers (114% higher in intervention compared to control) (*B* = 1.14, *P* < .001) and current use of modern contraceptives (57% higher) (*B* = 0.57, *P* < .01). All 13 community- and provider-developed indicators improved, with 6 of them showing significant improvements.

**Conclusions:**

By facilitating the relationship between community members, health service providers, and local government officials, the CSC contributed to important improvements in reproductive health-related outcomes. Further, the CSC builds mutual accountability, and ensures that solutions to problems are locally-relevant, locally-supported and feasible to implement.

## Introduction

Social accountability approaches have been growing in popularity in the health sector over the last decade. These approaches engage citizens in processes that strive to improve public sector performance and hold service providers and other actors accountable for delivering on their commitments [1]. Social accountability approaches aim to help service users voice their needs and concerns and hold service providers accountable for the provision of quality services. These approaches may be particularly effective at improving the patient-centered aspects of quality of care (for example, maintaining privacy and confidentiality, and providing respectful maternity care). Evidence suggests that a variety of social accountability approaches designed to achieve global maternal newborn health goals have improved community engagement in monitoring health services and increased service use, quality, and effectiveness [2, 3]. Although small in number, randomized controlled trials (RCTs) of social accountability approaches used in the health sector have demonstrated significant reductions in health provider absenteeism, and significant improvements in use of family planning and of health facilities for childbirth, attendance at prenatal care, child weight, and under-five child mortality [4–7]. The social accountability evidence base is limited, however, and results overall are still mixed [8–10]. A review of the mixed empirical evidence for social accountability argues that ‘more promising results emerge from studies of multi-pronged strategies that cultivate enabling environments for collective action and bolster state capacity to actually respond to citizen voice,’ but highlights the that both social accountability research and conceptual work are significantly lagging behind practice [8]. Therefore, there have been calls for additional evaluation research and evidence to ensure that these approaches ‘actually deliver benefits for women and children’ [2].

**Fig 1:**
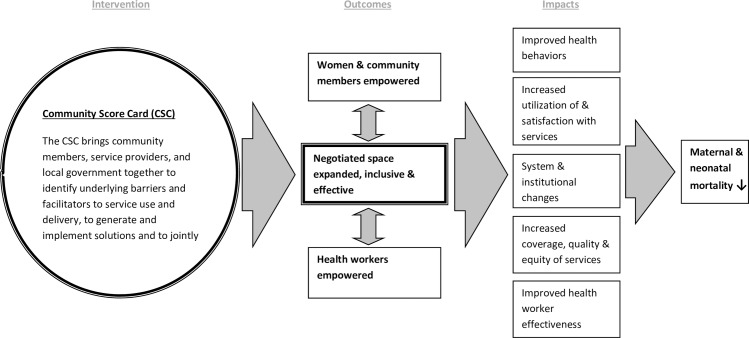
CARE’s Community Score Card Theory of Change

To this end, we designed a cluster-randomized control evaluation to assess the effectiveness of CARE’s Community Score Card (CSC) [11], a social accountability approach, to improve reproductive health-related outcomes in Ntcheu, Malawi. The theory of change underlying the CSC intervention (Fig 1) suggests that bringing together community members, health workers, and local officials to a) identify barriers and facilitators of service use and delivery, b) prioritize actions, and c) jointly monitor improvements will result in new and expanded spaces for inclusive, effective dialogue and negotiation. This, in turn, will empower both women and health workers in the community, leading to improved health behaviors, increased service utilization, and higher quality and more equitable service delivery. Ultimately, these changes, along with system and institutional changes, should decrease maternal and neonatal mortality in communities. Therefore, this evaluation aims to test the effectiveness of CARE’s CSC on maternal and reproductive health-related outcomes.

## Methods

### Summary of methods

CARE’s CSC was assessed in a cluster-randomized trial in the catchment areas of 20 health facilities the in Ntcheu district of Malawi. Health facilities were matched in pairs and one facility from each pair was randomly assigned to participate in the CSC and the other was assigned as a control health facility. Two-stage cluster sampling was used to select group villages and villages for participation in the study. Surveys were administered to 1301 women who had given birth within the last 12 months at baseline in November and December 2012, and 1300 women who had given birth within the last 12 months at endline in November and December 2014. Difference-in-difference and local average treatment effects were estimated to evaluate the CSC’s impact on modern contraceptive use, antenatal and postnatal care service utilization, and service satisfaction. Changes between scores in the first and last assessment of community- and service provider- developed Score Card indicators were compared.

### Study setting

Malawi is a small, landlocked country in southeast Africa heavily dependent on subsistence farming and fishing along Lake Malawi. Over 80% of the population lives in rural areas [12] and only 61% of the adult population is literate [13]. Despite tremendous improvements since 2000, Malawi still has relatively poor maternal and child health indicators. Overall life expectancy is only 55 years [13] with a total fertility rate of 5.4 [12]. Infant mortality is 45 per 1,000 live births [13] while the maternal mortality ratio is 510 per 100,000 live births [12]. The Ntcheu district in central Malawi lies half-way between the country’s two main cities of Blantyre and Lilongwe along the border with Mozambique.

Ntcheu has three hospitals and 33 health facilities administered by either the Malawi Ministry of Health (MOH) or the Christian Health Association of Malawi (CHAM). In 2011, 26 health facilities offered Prevention of Mother to Child Transmission of HIV (PMTCT) services, 6 provided basic emergency obstetric care (bEmOC), and 22 offered youth-friendly services. Family planning services are offered at all the MOH health facilities and some CHAM facilities. The CSC was evaluated via independent, cross-sectional baseline and endline surveys of women aged 15–49 who have given birth within the last 12 months whose baby was still living.

### Intervention description

The Community Score Card, developed by CARE Malawi in 2002 as a social accountability tool, aims to empower community members, health service providers, and other government officials to identify and overcome obstacles to health coverage, quality and equity in resource-limited settings [11]. In CARE’s experience, social accountability is a key strategy for empowering and supporting service users and service providers to work together to improve service delivery and outcomes. Working together builds awareness and understanding, as well as trust and sense of shared responsibility and motivation to act, which leads to improved responsiveness of the health system to the community’s needs [11].

**Fig 2:**
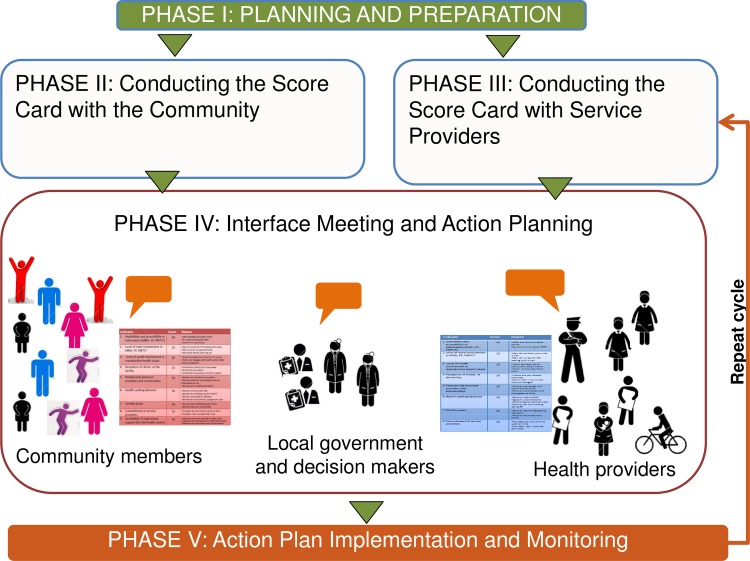
CARE’s Community Score Card Process (5 Phases).

The CSC intervention consists of five phases (see Fig 2). The first phase of the CSC intervention involves planning and preparation. This stage is critical and involves identifying the sectoral and geographic scope of the initiative, understanding the context and barriers both service providers and users face, training facilitators, and securing cooperation and buy-in from all participating parties, including government officials. In Phase 2, the CSC is conducted with the community via focus group discussions with community members (separated into groups such as men, women, youth, etc.) to identify and prioritize issues they are facing in accessing services. Identified issues are organized into themes and a measureable indicator is developed for each theme. The indicators are then verified and scored by the community, generating a Score Card. The community also indicates reasons for why a particular score was given and creates suggestions for improvement. The same process of issue generation and indicator development is conducted with service providers in Phase 3; through focus group discussions, service providers identify issues they are facing in delivering quality services, develop and score indicators, give reasons for the scores, and make suggestions for improvement. Phase 3 can occur either after or concurrently with Phase 2.

The CSC comes to life in Phase 4 at the interface meeting, during which community members and service providers are joined by local government officials and other power holders to share and discuss their respective Score Cards, issues and priorities. This joint conversation gives way to locally identified solutions and a community-wide action plan for service improvement. Finally, Phase 5 involves action plan implementation, monitoring, and evaluation in which community members, service providers, government staff and additional power-holders all have a role to play in reviewing and monitoring progress on indicators. This cycle is repeated (minus the initial planning and preparation stage) every six months: communities and service providers reconvene to discuss issues (and generate new ones, if needed), re-score the indicators and discuss reasons for changes, and then meet in an interface meeting to review their respective Score Cards, in an on-going cycle of problem identification, solution generation, implementation of improvements, and mutual accountability.

The CSC intervention evaluated here focused on maternal and reproductive health-related outcomes such as family planning, antenatal and postnatal care service utilization, and use of the health facility for labor and delivery. CARE Malawi facilitated Score Card processes with service providers- including both facility-based service providers and community health workers (CHWs), such as Health Surveillance Assistants (HSAs), and community members in the 10 intervention sites (see below for further description). Half of the intervention sites completed 4 cycles of the CSC process by the start of the endline data collection while the other half had completed 3 cycles. We hypothesized that the CSC would increase service utilization, perceptions of service quality, and satisfaction with services in the intervention communities.

### Study design

**Fig 3:**
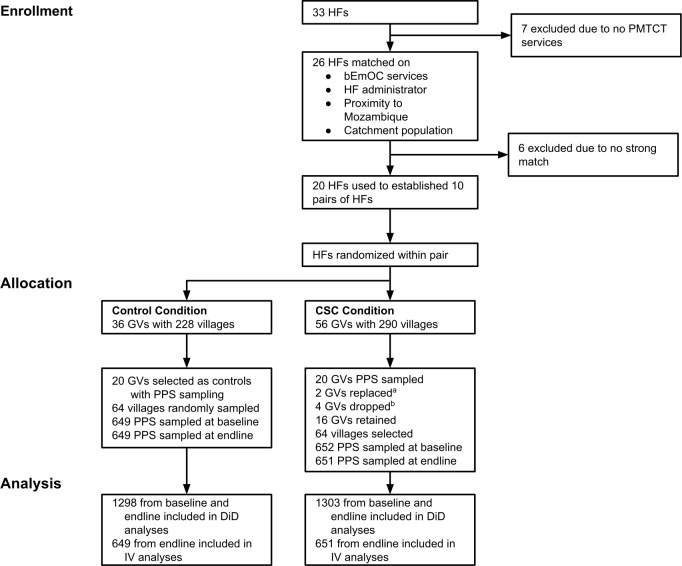
Randomization Design Flowchart. Footnotes: HF: health facility; GV: group village; PMTCT: Prevention of Mother to Child Transmission of HIV; bEmOC: basic emergency obstetric care. ^a^One of the selected treatment GVs consisted of a large number of individuals that used a different HF; another GV was affected by an external maternal and child health project. These two GVs were replaced with alternative GVs. ^b^One of the sampled HFs had eight GVs selected which was too many to feasibly implement the CSC for a single HF. Four GVs were eliminated and the PPS sample for this HF was obtained from the remaining four GVs.

Study sites were defined as a health facility and its surrounding catchment area; there were 33 health facilities and surrounding catchment areas in the initial population. We excluded 13 of the 33 available health facilities either because they did not provide PMTCT services or they did not have one or more of the required matching criteria. From the remaining health facilities, we created matched pairs using the following characteristics: presence of bEmOC services, who administered the facility (MOH or CHAM), proximity to the Mozambique border (as this had implications for the population using the facility as well as for the ability of health workers to provide services), and population size of the catchment area. This process resulted in ten matched pairs of health facilities. From each pair, we randomly assigned one health facility to the intervention condition, and the other was assigned to the control condition (see Fig 3).

We used data obtained from government census, district, and local office sources to construct the population from which we would draw the sample. Among intervention health facilities, there were 56 group villages (GVs) that contained 290 villages with a total population of 228,029. Among control health facilities, there were 36 GVs that contained 228 villages with a total population of 170,201. Using UNICEF’s probability-proportional-to-size (PPS) sampling method [14], we selected twenty GVs (i.e., clusters) from the intervention area and twenty GVs in the control area to serve as the primary sampling units. One of the largest intervention health facilities contained eight sampled GVs. Because we could not feasibly implement the CSC for all eight GVs in a single health facility, four GVs were dropped (leaving 16 GVs in the sample), and the PPS sample for this health facility was obtained from the remaining four GVs. The CARE Malawi team purposively identified 64 villages from the 16 intervention GVs in which to work, and randomly selected 64 villages in the 20 control GVs; the same PPS method described above was used to select villages. The number of individuals sampled in each village was determined by number of eligible women in a village multiplied by the sampling proportion for the condition (i.e., the required sample size divided by the total eligible population). We sized the sample to detect a 10% change in institutional births, based on the prevailing rates of institutional births in Ntcheu (78%) [15], prior to baseline. Given the hypothesized effect size, our power analysis determined a sample of 650 women per treatment condition (power = .80, 2-tailed α = .05, non-response = 5%, and design effect = 2.0).

### Data collection

We collected baseline data (n = 1301) between November and December 2012, and endline data (n = 1300) in November and December 2014. We selected every third household starting with a random spin of a bottle near the center of the village and working outward. When there was more than one eligible respondent in the household, data collectors randomly chose a person to be interviewed using a Kish grid. This process was repeated until the criterion sample size for the village had been met. When the sample size could not be met, interviewers went to the nearest village to complete data collection. The survey took 40–60 minutes to complete; all data were self-reported. All women provided verbal informed consent prior to the start of the survey. The study was reviewed by Malawi’s National Health Science Research Committee and deemed a program evaluation.

### Measures

#### Outcomes

We evaluated a variety of service utilization, perceived service quality, health behavior and supportive care outcomes. We defined use of modern family planning as women who were not pregnant and reported currently using female or male sterilization, oral contraceptive pills, an intrauterine device, injectable contraception, implants, or male or female condoms. We evaluated three pregnancy-related service utilization outcomes: early antenatal care (ANC)–women who received their first ANC visit within the first 3 months of pregnancy; sufficient ANC–women who received at least 4 ANC visits; and CHW home visit during last pregnancy. Postnatal service utilization outcomes included receiving any check-up within 2 months after the baby was born, receiving 2 or more check-ups, and receiving a CHW home visit during the postnatal period.

For perceived service quality, we tested perceptions of both specific service quality and overall service satisfaction. We measured perceived quality of services for ANC, skilled delivery care, postnatal care, and birth planning using multi-item indexes. For quality of family planning, we assessed the counseling environment and the clarity of explanation via separate indexes. See Table 1 for a full description of the indicators comprising the composite variables.

**Table 1:** Description of the indicators comprising the service satisfaction, service quality and birth planning indexes.

**Satisfaction with services: Index of maternal health services received**
How satisfied were you with the overall quality of …
•… care you received during your ANC visit(s)? •… care you received during your labour and delivery at [health facility]? •… HIV/AIDS information, services, and care you received during pregnancy and delivery? •… family planning services that you received? •… you and/or your baby received during your post-partum visits?
**Family planning (FP): Index of FP service quality provision**
Thinking about the last time you received family planning services, …
•… did your partner, family or anyone else try to discourage you from using family planning? (reversed) •… did anyone at the health facility discourage you from trying to use family planning? (reversed) •… did anyone at the clinic make you feel embarrassed or ashamed of asking for family planning services? (reversed) •… were you treated with respect and dignity? •… were you treated with kindness and understanding? •… were you given all the information or explanations you needed? •… were you spoken to in a way that you could understand? •… did the provider talk to you about how long you would like to wait before having another child? •… did the health provider tell you that it was your decision whether you choose to use family planning? •… did the health provider ask if you wanted your partner to join in the discussions about family planning? •… did you feel the information you shared during your visit would be kept confidential? •… overall, did you feel it was your decision alone whether to use family planning?
**Family planning: Index of FP service quality provision**
**For the method you got, did the health provider …**
•… explain how to use the method effectively •… describe possible side effects •… tell you if the method would protect against HIV •… tell you when you should return for a follow-up visit
**Antenatal care: Index of ANC service quality**
During your pregnancy, did a health provider or HSA talk to you about the following:
•Danger signs during pregnancy and childbirth? •The importance of going to a health facility for antenatal checks? •The importance of HIV testing during pregnancy? •How to create a birth plan to prepare for the birth of your child? •How to delay or prevent another pregnancy after your delivery? •The importance of exclusive breastfeeding?
**Delivery care: Index of delivery service quality provision**
Thinking about your care during labour and childbirth …
•… were you able to move around and choose the position that made you most comfortable? •… did you feel you got the pain relief you wanted? •… was the labour or delivery room you were in clean? •… did you have confidence and trust in the staff caring for you during your labour and childbirth? •… were you (and/or your partner or a companion) left alone by midwives or doctors at a time when it worried you? (reversed) •… were you given the information or explanations you needed? •… were you spoken to in a way you could understand? •… were you treated with respect and dignity? •… were you treated with kindness and understanding? •… were you involved enough in decisions about your care? •… did the health provider yell at or humiliate you in any way? (reversed) •… did you receive an injection immediately after your baby was born? •… did the health provider check on you and your baby for any problems prior to discharge?
**Postnatal care: Index of PNC service quality provision**
During these services after your baby was born, did a health worker do any of the following?
•Counsel you on danger signs to watch for in you and in your child? •Give you breastfeeding support and counseling? •Counsel you on methods to avoid or delay another pregnancy? •Provide you HIV counseling?
**Birth planning: Index of birth planning**
**During your most recent pregnancy, did you do any of the following to prepare for the birth of your child?**
•Decide which health facility you would go to for delivery? •Arrange for the means of transport you would use when going to the health facility for delivery? •Arrange for someone to accompany you to the health facility during delivery?

To measure overall service satisfaction, we asked respondents how satisfied (completely unsatisfied to completely satisfied) they were with quality of care during ANC visit(s), labor and delivery, HIV/AIDS information and services, FP services, and postnatal visits. We used exploratory factor analysis [16] to evaluate factor structure, and determined that a single factor was sufficient for summarizing the five items. Thus, the items were averaged and treated as a general satisfaction construct (α = .67). For supportive care, we measured partner presence during any ANC visit and for HIV-testing.

#### Covariates

We controlled for religion (Catholic vs. other), ethnicity (Ngoni vs. other), current marital status (married/living together vs. other), literacy (reads full sentence vs. other), number of lifetime live births, time to closest place to give birth (less than 30 minutes, 30–59 minutes, 1–2 hours, and over 2 hours), and wealth index in all models. The wealth index was constructed using a subset of indicators drawn from the Demographic and Health Surveys (DHS) methodology [17].

#### Score Card

Score Card indicators were developed based on the issues raised during the Score Card process. Community members and service providers developed 12 indicators to track progress, for example, reception of clients at the facility, level of male involvement in maternal newborn health (MNH) issues, and availability of transportation for referrals during labor and delivery. CSC participants and service providers generated similar issues, but from their different perspectives. For example, “relationship with providers” was an indicator for both: from the community side this referred to how providers treated them, whereas from the provider’s side, it referred to things like patients not listening to them, or following their guidance. The service providers also generated one additional indicator—availability of supervisory support—for a total of 13 Score Card indicators. In an open discussion, participants agreed on scores for each indicator using a scale from 0–100. This was done with the communities and the service providers separately, and then, during the interface meeting the Score Cards were discussed and actions to improve scores were agreed upon. For each intervention site, there were 1–4 community Score Cards and 1 service provider Score Card. The same indicators were used across all 10 intervention sites and were re-scored during each 6 month CSC cycle.

### Analysis

#### Survey analysis

Prior to fitting hypothesis-testing models, we checked for balance among our demographic covariates across the two conditions, using a Rao-Scott Χ^2^ test for categorical variables and simple regression for continuous variables.

We then used two modeling strategies to investigate the impact of the CSC on outcomes. First, **difference-in-differences** (DiD) models allowed us to estimate the differential change in the treatment versus control areas over time [18, 19]. This method estimates differential change for repeated cross-sections by including a time, treatment, and time-by-treatment interaction term in the model. Obtaining a significant and positive time-by-treatment interaction indicates a greater change from baseline-to-endline for the treatment areas versus the control areas.

Second, we used **local average treatment effect** (LATE) models [20] to estimate CSC impact among respondents who participated in the CSC process at endline, because direct participation in the intervention was relatively low. Only 25.8% of surveyed women in the intervention villages reported having participated in the CSC by endline; while in the control villages, 5.7% of surveyed women indicated participation (suggesting treatment leakage). Given these rates, a typical intent-to-treat analysis would underestimate program effects. The LATE approach provides an unbiased estimate of the theoretical impact of the intervention had 100% of the treatment group participated and there was no treatment leakage to the control group [20]. LATE estimates were obtained in separate models for each of the outcomes. All models contained CSC participation instrumented on treatment assignment and the covariates described above.

All analyses utilized weighted data proportional to sampling probability with village, GV, and health facility cluster weights; standard errors were computed using SAS 9.3 and STATA 14 procedures to account for the complex sampling design. Nonlinear relationships were tested using quadratic terms for continuous predictors and retained if significant. Linear regression estimates are presented for binary outcomes due to known limitations with generalized linear models in both the DiD (Lechner, 2010) [21] and LATE (Angrist & Pischke, 2009) [19] frameworks.

#### Score Card analysis

We also examined changes in the scores on the Score Card over time. For our analysis, we averaged the scores across the ten intervention sites and compared the first and the last scores for each indicator, using a Z test of statistical significance.

## Results

### Survey results

We found no significant differences in socio-demographic characteristics between intervention and control areas at baseline: religion (Χ^2^[1] = 0.17, *P* = .69), ethnicity (Χ^2^[1] = 0.16, *P* = .69), current marital status (Χ^2^[1] = 0.22, *P* = .64), literacy (Χ^2^[1] = 2.53, *P* = .11), number of lifetime live births (*B* = 0.09, 95% CI -0.20–0.38, *P* = .53), time to closest place to give birth (Χ^2^[3] = 2.25, *P* = .52), and wealth index (*B* = -0.15, 95% CI -0.54–0.24, *P* = .43), suggesting that our matched-randomized design successfully balanced these dimensions (see Table 2 for a summary of demographic characteristics).

**Table 2:** Selected socio-demographic and household characteristics of women who gave birth in the last 12 months: Baseline, 2012^1^.

Characteristic	Controls	Intervention
	N = 649	N = 652
Age (years) (%)		
15–19	18.7%	17.3%
20–24	30.5%	30.9%
25–29	26.9%	24.1%
30–34	15.1%	17.8%
35–45	8.8%	9.9%
Religion (%)		
Catholic	21.6%	25.3%
Presbyterian	12.1%	14.2%
Other Christian	62.0%	56.6%
Other	4.3%	4.0%
Ethnicity (%)		
Ngoni	89.6%	88.4%
Other	10.4%	11.6%
Marital Status (%)		
Never married & never lived together	6.3%	3.1%
Married/currently living together	88.2%	89.2%
Divorced/separated/widowed	5.5%	7.7%
Reading level (%)		
Cannot read simple sentence	29.0%	29.3%
Can read part of the sentence	9.1%	15.1%
Can read the entire sentence	61.9%	55.6%
Number of live births (%)		
1	26.9%	26.2%
2	20.1%	21.1%
3–4	34.9%	31.5%
5+	18.2%	21.2%
Time to reach the closest place to give birth (%)		
Less than 30 minutes	11.1%	16.3%
30–59 minutes	30.3%	32.1%
1–2 hours	38.9%	36.7%
More than 2 hours	19.7%	14.9%
Household wealth (mean/SE)^2^	0.14 (0.16)	-0.01 (0.10)
Greater than 1 acre of land^3^	49.6%	53.1%
Metal roof^3^	16.9%	14.7%
Electricity, solar power or generator^3^	3.7%	3.2%

Footnotes

^1^weighted percentages & means.

^2^computed from principal components analysis implemented using the Demographic and Health Surveys (DHS) methodology (Rutstein & Johnson, 2004).

^3^One of 12 indicators used to calculate the wealth index.

A substantial proportion of our sample was under the age of 20 (18%) and approximately half of the mothers were under 25. More than 50% of the sample had 3 or more children reflecting the country’s high fertility rate. Almost 90% of the women were married or living with their partner. Like Malawi overall, a significant minority of the sample was Catholic, but more than three quarters were non-Catholic Christians. Also, like Malawi overall, only two-thirds were literate. Ntcheu is dominated by the Ngoni ethnic group as was our sample. More than 50% of the women reported living an hour or more from the closest health facility.

Several of our outcome variables were reported as very high across the sample prior to intervention (see Table 3). For example, women reported near universal ANC utilization during the last pregnancy (99.2%), delivery in a health facility (97.2%), skilled delivery (95.0%), HIV testing during pregnancy (92.8%), and breastfeeding (100%) at baseline. On the other hand, rates of reported visits by health workers were quite low, especially for postnatal care. A little over 50% of women reported currently using modern contraception, and approximately one third said their partner had accompanied them for an antenatal visit, and over one third had gone with them for HIV testing. The high baseline rates limited the number of outcomes for which we could evaluate change from the CSC intervention. Because there was insufficient room for improvement, we excluded outcomes that were at 90% or greater at baseline, and we eliminated three continuous outcomes—indices of antenatal and postnatal care quality, and of birth planning—because they were within 0.50 standard deviations of their maximum value at baseline.

**Table 3:** Selected maternal health characteristics among women who gave birth in the last 12 months: Baseline, 2012^1^.

	Indicator	%
**Maternal health service utilization**		
Family planning	Currently using modern FP	53.5%
	Current use	
	Injectables	40.2%
	Other modern methods^2^	13.2%
	Traditional methods^3^	0.2%
Antenatal care	Antenatal care use at last pregnancy	99.2%
	Early ANC	16.4%
	Sufficient ANC received (4+ visits)	53.0%
	Number of times that antenatal care was received (mean (SE; [range]))	3.67 (0.06; [0, 10])
	Visited by a community health worker during last pregnancy	18.3%
Delivery care	Last delivery occurred in a health facility	97.2%
	Last delivery with skilled personnel	95.0%
Postnatal care	Postnatal care use at last pregnancy	76.4%
	Postnatal care within 24 hours	16.8%
	Number of checks within 2 months postpartum^a^ (mean (SE; [range]))	1.21 (0.06; [0, 18])
	Visited by a community health worker postpartum	5.2%
HIV testing	HIV testing during pregnancy	92.8%
**Perceived quality of services when last received**		
Satisfaction with services	Index of maternal health services received (mean (SE; [range]))	4.82 (0.02; [3, 5])
Family planning	Index of FP service quality provision^4^^,^ ^b^ (mean (SE; [range]))	11.61 (0.03; [6, 12])
Family planning 2	Index of FP service 2 quality provision^5^^,^ ^c^ (mean (SE; [range]))	3.48 (0.04; [0, 4])
Antenatal care	Index of ANC service quality (mean (SE; [range]))	5.77 (0.03; [0, 6])
Delivery care	Index of delivery service quality provision^6^^,^ ^d^ (mean (SE; [range]))	11.31 (0.08; [0, 13])
Postnatal care	Index of PNC service quality provision^7^^,^^a^ (mean (SE; [range]))	3.80 (0.02; [0, 4])
**Health behaviors**		
Birth planning	Birth planning index^8^ (mean (SE; [range]))	2.75 (0.02; [0, 3])
Breastfeeding	Any	100.0%
	Within 24 hours after delivery	97.9%
**Supportive care**		
Male involvement	Husband/partner present during any ANC visit^e^	32.2%
	Went for HIV testing with husband/partner^f^	38.3%

Footnotes

^1^weighted percentages & means

^2^includes female or male sterilization, oral pills, intrauterine device, implant, & male or female condoms

^3^includes standard days/rhythm, abstinence, withdrawal, & breastfeeding

^4^ constructed using 12 items that assessed whether respondents were discouraged, treated with respect and understanding, given explanations, the mother’s decision was emphasized, and confidentiality was emphasized in discussions of family planning (range 0–12).

^5^constructed using 4 items: provider explained how to use chosen FP method, explained possible side effects, mentioned if method protects against HIV, & scheduled follow-up (range 0–4)

^6^constructed using 5 items: able to move around & choose the position that made her most comfortable, got the pain relief she wanted, not left alone by providers at a time when it worried her, provider(s) did not yell or humiliate the respondent in any way, & respondent felt involved in decision about her care (range 0–5)

^7^constructed using 4 items: health worker provided counsel on danger signs in mother and child, breastfeeding support and counseling, counsel on methods to avoid or delay pregnancy, and you HIV counseling (range 0–4)

^8^constructed using 3 items: during last pregnancy, women decided where to deliver, arranged transportation to get to the facility, and arranged for a companion to accompany her to facility (range 0–3)

^a^Only asked of respondents who indicated that they someone had checked on their baby within two months after the baby was born (*n* = 1005).

^b^Only asked of respondents who indicated that they had ever received family planning services (*n* = 947).

^c^Only asked of respondents who indicated that they had ever received family planning services and were chose a modern family planning method (*n* = 922).

^d^Only asked of respondents who indicated that they had delivered in a hospital or health facility (*n* = 1268).

^e^Only asked of respondents who indicated that they had seen someone for antenatal care (*n* = 1291).

^f^Only asked of respondents who indicated that they had ever been tested for HIV (*n* = 1226).

DiD analyses showed a 20% greater increase in the number of women receiving a CHW home visit during most recent pregnancy in the intervention versus the control villages (*B* = 0.20, 95% CI 0.07–0.33, *P* < .01), as well as a 6% greater increase in the number of women receiving a CHW postnatal visit (*B* = 0.06, 95% CI 0.01–0.10, *P* = .01) (see Table 4). Overall service satisfaction ratings also improved significantly more in intervention villages (*B* = 0.16, 95% CI 0.07–0.24, *P* < .001). No other outcomes showed significant differential change over time between the intervention and control villages.

**Table 4:** CSC impact on selected outcomes among women who gave birth in the last 12 months: Difference-in-differences (DiD) estimates.

	Outcome	DiD	95% CI	*t*	*P*
**Maternal health service utilization**					
Family planning	Currently using modern FP	0.05	-0.07–0.16	0.81	.42
Antenatal care	Early ANC	-0.03	-0.11–0.05	-0.65	.52
	Sufficient ANC received	0.04	-0.11–0.18	0.51	.61
	Visited by a community health worker during last pregnancy	0.20	0.07–0.33	3.12	< .01
Postnatal care	Postnatal care use at last pregnancy	0.02	-0.11–0.15	0.34	.74
	Postnatal care by a community health worker	0.06	0.01–0.10	2.56	.01
**Perceived quality of services when last received**					
	Satisfaction with services	0.16	0.07–0.24	3.66	< .001
	Quality of the FP counseling environment	0.12	-0.11–0.34	1.05	.30
	Clarity of FP explanations	-0.16	-0.38–0.06	-1.48	.14
	Delivery care	0.44	-0.04–0.93	1.81	.08
**Supportive care**					
	Husband/partner present during any ANC visit	-0.10	-0.29–0.08	-1.10	.28
	Went for HIV testing with husband/partner	-0.04	-0.23–0.14	-0.47	.64

The LATE estimates (see Table 5) indicated that a significantly higher proportion of pregnant women received a CHW home visit during pregnancy in the intervention area as compared to the control area (*B* = 1.14, 95% CI 0.61–1.68, *P* < .001). Furthermore, we estimated a 57% greater current use of modern family planning in the intervention area (*B* = 0.57, 95% CI 0.17–0.96, *P* < .01). No other outcomes appeared to be different between intervention and control areas at endline.

**Table 5:** CSC impact on selected outcomes among women who gave birth in the last 12 months: Local average treatment effect (LATE) estimates, endline, 2014.

	Outcome	LATE	95% CI	*t*	*P*
**Maternal health service utilization**					
Family planning	Currently using modern FP	0.57	0.17–0.96	2.90	< .01
Antenatal care	Early ANC	0.08	-0.21–0.37	0.57	.57
	Sufficient ANC received	0.43	-0.20–1.06	1.40	.17
	Visited by a community health worker during last pregnancy	1.14	0.61–1.68	4.32	< .001
Postnatal care	Postnatal care use at last pregnancy	-0.22	-0.64–0.20	-1.05	.30
	Postnatal care by a community health worker	0.14	-0.02–0.30	1.79	.08
**Perceived quality of services when last received**					
	Satisfaction with services	0.24	-0.05–0.54	1.67	.11
	Quality of the FP counseling environment	0.32	-0.62–1.26	0.68	.50
	Clarity of FP explanations	0.24	-0.48–0.96	0.68	.50
	Delivery care	0.98	-0.90–2.87	1.06	.30
**Supportive care**					
	Husband/partner present during any ANC visit	-0.11	-0.73–0.52	-0.34	.74
	Went for HIV testing with husband/partner	-0.27	-0.96–0.42	-0.80	.43

### Score Card results

**Fig 4:**
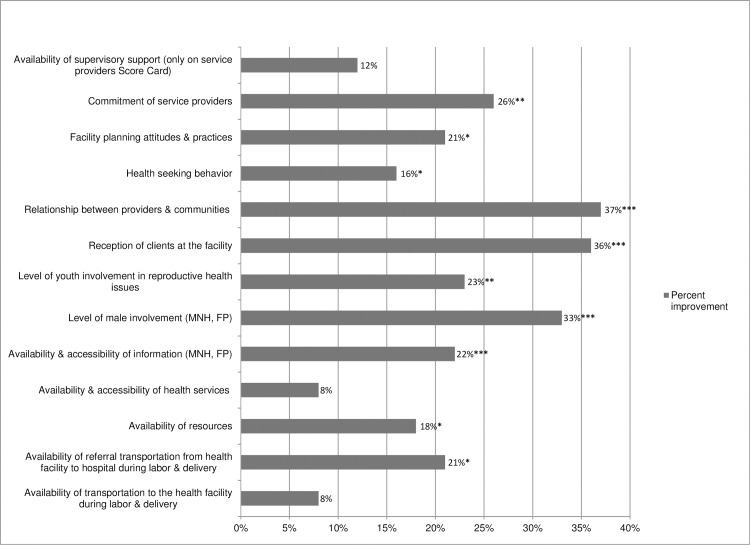
Change in Score Card Indicators from First to Final Scoring. Footnotes: †Z-test comparing the significance of 2 proportions (one-tailed p-value). *p-value significant at ≤.10; **p-value significant at ≤.05; ***p-value significant at ≤.01, MHN, maternal newborn health; FP, family planning.

We found improvement in all 13 CSC indicator scores between the first and final rounds of the Score Card, many quite substantial and statistically significant (see Fig 4). Relationship between health workers and communities and reception of clients at the facility saw the greatest increases with 37 and 36 point increases, respectively. Commitment of health workers gained 26 points. Other indicators with substantial increases included level of male involvement in maternal newborn health and family planning (33 points), level of youth involvement (23 points), and availability and accessibility of information (22 points).

## Discussion

This is the first study to use a rigorous cluster-randomized controlled design to evaluate the effectiveness of CARE’s CSC on a wide range of reproductive health-related outcomes. We found that in rural Malawi our CSC intervention increased CHW visits to women during pregnancy by 20% and during the postnatal period by 6%, compared to control. Further, women’s satisfaction with reproductive health services increased significantly, compared with control areas. In addition to these outcomes, our LATE analysis suggests the CSC also had a significant effect on use of modern contraception, with an estimated 57% greater use in the intervention versus control condition at endline. The 13 CSC indicators developed by community members and health providers to drive reproductive health progress also improved, many significantly, providing additional insight into how the CSC may have affected outcomes.

In several countries, CHWs have been identified as playing a critical role in improving universal health coverage [22]; however, there are a dearth of studies on the effectiveness of specific strategies to improve the functioning of CHW programs [23]. Our study fills this critical gap, and demonstrates that the CSC can be an effective strategy for increasing CHW home visits during both pregnancy and the postnatal period.

CHW programs are often plagued by insufficient community involvement and weak linkages to the formal health system, and institutionalizing and mainstreaming community participation and strengthening links with the formal health system pose key challenges [23]. Research also suggests that relationships within the community enable CHWs to optimally engage with community actors, promoting healthy behavior [24–27]. A study from Malawi suggests that in order to optimize HSA performance ‘there is a need to improve support and accountability structures that facilitate communication and dialogue, increase trust and manage expectations and thereby improve interpersonal relationships between HSAs and actors in the community and health sector’ [28].

The CSC provides a concrete approach to tackling these key issues, and the increases in the CSC indicators suggest that it is effective in doing so. For example, there was a significant 37% increase in the CSC indicator on the relationship between the community and health providers, as well as 22% increase in the indicator on availability and accessibility of reproductive and maternal health information. CHWs were pivotal to driving improvement in this CSC indicator and various actions were taken to equip and support them to do so- for example, the district government supported CHW capacity building and community members mobilized resources to build CHW’s houses and clinics. Inadequate supervision has also been raised as a challenge to a well-functioning CHW program [29], and again, we saw improvement (12%, non-significant) in the CSC indicator on the availability of supervisory support.

The CSC’s impact on women’s satisfaction with reproductive health services is promising since patient satisfaction has been shown to be an important predictor of positive patient behaviors such as service utilization [30]. Patient satisfaction is affected by many variables. One study that conducted factor analysis of key variables showed two primary factors at work: provider performance—especially as it relates to interpersonal relations and patient-caregiver interactions (responsiveness, dependability, empathy, caring); and access—variables related to the patient’s ability to gain care and the impediments to that process [31].

As the improvements in CSC indicators suggest, these are precisely the variables that the CSC can address—improving communication, trust, responsiveness and quality of patient-provider interactions—and overcoming barriers to accessing services by generating locally-relevant solutions to improving access. Three CSC indicators of the relationship between health workers and communities improved substantially—how clients are received at facilities, the relationship between health providers and the community, and commitment of service providers. Furthermore, all 13 indicators—identified by the community and service providers as critical to improving reproductive health services access, utilization and quality provision- all improved through the deployment of locally developed solutions. There were significant improvements in CSC indicators that could be resolved at the community and health provider level, with little or no resources required from the government, whereas the 3 CSC indicators that did not improve significantly were those that hinged on responses from the district government or higher, and required resources (e.g. supervision visits require vehicles and fuel for vehicles).

Though we hypothesized an increase in satisfaction with services, we were initially concerned about the potential for decreased satisfaction as community members became more aware of “what they were missing”, as they became more knowledgeable and involved in the governance of local health services. Instead, as predicted, we saw an improvement in overall satisfaction with services and a decline in the control communities. The decline in the control communities may reflect frustration with a widespread and prolonged stock-out of commodities resulting from a disruption in donor funding to Malawi during the ‘Cashgate’ scandal [32]. The CSC intervention might have buffered the intervention communities from this decline in satisfaction; in addition to improving communication and trust, these communities may have developed a deeper appreciation for the complexities of delivering health services at the local level, and an understanding of what local officials can and cannot control directly.

The large increase in use of modern contraception indicated by our analysis, is of particular promise. Family planning is a cornerstone of development [33], and ensuring equitable access to high quality family planning information, services and supplies has re-merged as a global focus since the Family Planning Summit in London in 2012. For Malawi, reducing adolescent pregnancy, and helping adolescents, women and couples plan if, when and how many children to have is an urgent priority [34]. There is also an increasing recognition of the potential of local level social accountability approaches in ensuring that family planning programs respect, protect and fulfill individuals’ rights to full, free and informed choice and quality, non-discriminatory care [35].

Our study is one of only a few RCTs that focuses specifically on the potential benefits of a social accountability approach, like the CSC, on improving access to, and use of, family planning services. The Bjorkman study in Uganda (Bjorkman and Svensson2009) [4] used a similar social accountability approach, and showed an increase of 22% in use of family planning after just one year. Taken together, these results suggest that contraceptive use may be particularly sensitive to these kinds of approaches—establishing trust and improving patient-provider relationships, as well as identifying and successfully addressing local level bottlenecks, may be two critical factors in enabling increased uptake of family planning services. The significant CSC-catalyzed increases in the level of male involvement in MNH and FP, level of youth involvement in reproductive health issues, relationship between providers and communities, and availability and accessibility of information (all shown in the CSC indicator results) may have contributed to the large increase in use of modern contraceptives.

### Limitations

We did not see improvements for as many outcomes as we expected in the intervention areas, in part because high baseline levels of several outcomes left little room for improvement. The self-reported levels of skilled delivery care, institutional delivery, and breastfeeding in our baseline survey were substantially higher than had been reported from the same district by the 2010 Malawi DHS [15], suggesting the potential for social desirability bias in our survey. Furthermore the Government of Malawi encouraged institutional delivery in order to reduce maternal mortality rates, by prohibiting use of traditional birth attendants and penalizing women who do not deliver at a health facility. Thus women may have felt significant pressure to report delivery in a health facility.

Second, we selected our intervention area partly because few other non-governmental organizations were working on maternal and child health in the district. However during the intervention period, a number of organizations began to show interest in the district, threatening to contaminate the evaluation design. Neither we nor the government wanted to hinder investment in maternal and child health in the district, so we shortened the evaluation period and conducted the endline after two rather than three years of intervention. This shortened timeline reduced the intervention dose—number of Score Card cycles completed in each community–and decreased the timeframe in which to observe effects.

Third, there was a major disruption of donor funds to Malawi during the study period; Cashgate interrupted supply chains and caused stock-outs of health commodities. While the CSC is designed to increase transparency and accountability of health services at the local level, it is limited in its ability to affect national and international issues.

Finally, eight GVs were selected from one of the largest health facilities and four of those were dropped because we could not feasibly implement the CSC in all eight of the selected GVs. The resulting imbalance in the number of GVs in the control and CSC conditions, potentially reduced the power of statistical models. Despite these various challenges, we were still able to implement and evaluate the CSC in a cluster-randomized nature as designed and saw improvements in several important health-related outcomes in a relatively short time-period.

## Conclusions

Increasing evidence suggests that social accountability interventions like the CSC are an effective way to improve maternal and reproductive health services and outcomes in low-resource settings. One of the greatest strengths of the CSC process may be that it helps build understanding and a stronger, more trusting relationship between the health system and the community. By getting both community members and frontline health providers involved and invested in governance over local health services, a new dynamic of working collectively to overcome challenges and improve outcomes is established.

Sustained improvements in coverage, quality and equity of services can only be achieved by shared responsibility and accountability for outcomes among key stakeholders. The CSC strives to improve stakeholder interactions by collaboratively engaging community members and service providers. Interface meetings provide a safe space where constituents can share concerns, think through solutions, and negotiate joint action plans. Our results demonstrate that this activity can enhance patient-centered care, community engagement, ongoing feedback, and availability of information about services.

Most barriers to implementing quality services can be best identified and addressed on the front lines. Too often, policy makers and program developers devise “innovative solutions” apart from frontline stakeholders, and then struggle to persuade communities and staff to adopt them. The CSC gives those with the most to gain and lose the tools to ensure that solutions are locally supported, relevant, and feasible. By improving the responsiveness of the health system to the self-identified needs of the population it serves, the CSC seeks to fundamentally change the relationship between the community and the health delivery system, ensuring that they work together as a complete system to improve maternal newborn health.

## Supporting information

S1 File Data. (XLS)
